# Characterization of prophages of *Lactococcus garvieae*

**DOI:** 10.1038/s41598-017-02038-y

**Published:** 2017-05-12

**Authors:** Giovanni Eraclio, Maria G. Fortina, Simon J. Labrie, Denise M. Tremblay, Sylvain Moineau

**Affiliations:** 1 Department of Food, Environmental and Nutritional Sciences, Division of Food Microbiology and Bioprocesses, University of Milan, Via Celoria 2, 20133 Milan, Italy; 2 GREB & Félix d’Hérelle Reference Center for Bacterial Viruses, Faculté de médecine dentaire, Université Laval, Québec City, Québec G1V 0A6 Canada; 3 Département de biochimie, de microbiologie et de bio-informatique & PROTEO, Faculté des sciences et de génie, Université Laval, Québec City, Québec G1V 0A6 Canada

## Abstract

This report describes the morphological characterization and genome analysis of an induced prophage (PLg-TB25) from a dairy strain of *Lactococcus garvieae*. The phage belongs to the *Siphoviridae* family and its morphology is typical of other lactococcal phages. A general analysis of its genome did not reveal similarities with other lactococcal phage genomes, confirming its novelty. However, similarities were found between genes of its morphogenesis cluster and genes of Gram-positive bacteria, suggesting that this phage genome resulted from recombination events that took place in a heterogeneous microbial environment. An *in silico* search for other prophages in 16 *L. garvieae* genomes available in public databases, uncovered eight seemingly complete prophages in strains isolated from dairy and fish niches. Genome analyses of these prophages revealed three novel *L. garvieae* phages. The remaining prophages had homology to phages of *Lactococcus lactis* (P335 group) suggesting a close relationship between these lactococcal species. The similarity in GC content of *L*. *garvieae* prophages to the genomes of *L. lactis* phages further supports the hypothesis that these phages likely originated from the same ancestor.

## Introduction

Bacterial viruses (phages) are considered the most abundant and diverse biological entities in our biosphere. Yet, most phages can be classified into just two main categories. Virulent phages can only replicate through a lytic cycle, which leads to cell lysis and the release of new virions ready to infect other sensitive hosts. On the other hand, temperate phages also have the ability to complete a lysogenic cycle in which they integrate their genome into the bacterial host chromosome and, thereby, replicate with the genome of the cell. Once the genome of a temperate phage is injected into the cytoplasm of its bacterial host, depending on the metabolic state of the cell, the phage genes involved in the lysogenic cycle may be expressed to favour this lifestyle. The lysogeny state will be maintained until stress conditions cause activation of the prophage through the transcription of lytic genes and the beginning of the lytic cycle^1, 2^.

The number of studies on phage-bacteria interactions has increased in the past decade. Many of these studies are related to a revival in the potential use of lytic phages as alternatives to antimicrobials for a myriad of applications, including inactivating antibiotic-resistant bacterial pathogens^3^. There has also been an increased interest in further understanding the defense mechanisms used by bacteria to combat phages^4, 5^.

Studies on temperate phages have mostly focused on their ability to contribute to bacterial evolution and adaptation to different environments rather than on their antimicrobial activities. Generally, prophages are responsible for changing the host’s behaviour, including granting immunity against infection by the same or closely related phages, disrupting bacterial gene(s) during genome integration, as well as modulating host gene expression through phage promoters. In some cases, temperate phages carry genes coding for toxins, regulatory and effector proteins, adhesins, exonucleases and superantigens. These sequences are often flanked by a specific transcriptional promoter and terminator, allowing gene expression during the lysogenic cycle^6^. The presence of a new prophage in a bacterial strain may lead to the so-called “lysogenic conversion”, where a non-pathogenic strain is converted into a pathogen by the integration of a temperate phage genome carrying genes coding for toxin or virulence factors. One well-characterized example is *Escherichia coli* O157:H7, where new clones have emerged following the acquisition of two Shiga toxin-encoding prophages (Sp5 and Sp15)^7^. Strains of *Vibrio cholera* have also acquired the cholera toxin through a filamentous phage^8^.

*Lactococcus garvieae* is one of the most important pathogens in the aquaculture sector^9^. This bacterial species is also found in different food matrices^10^. In addition, clinical cases associated with *L. garvieae* infection, albeit rare, have been reported in humans^11^. Little information is currently available about the pathogenic potential of *L. garvieae*. Much is known about its evolutionary history and ability to colonize diverse environments, and *L. garvieae* and the industrially-relevant dairy species *Lactococcus lactis* have a phylogenetic relationship, with more than 900 genes in common^12^. Analysis of its Mobile Genetic Elements (MGEs) showed a high degree of variability that can be linked to the lifestyle of this species. In particular, the distribution of insertion sequences (IS) has been used to characterize different ecotypes^13^. Finally, not much is known about phages infecting *L. garvieae*. To our knowledge, only two virulent phages of *L. garvieae* have been described and studied at the genomic level^14, 15^, and more recently, a temperate *L. garvieae* phage induced from a strain isolated from a marine fish in Japan was characterized^16^.

Here, we describe the isolation and characterization of a new temperate phage from a dairy strain of *L. garvieae*. We also searched for prophages in 16 *L. garvieae* genomes available in public databases, leading to the identification of three novel prophages.

## Results

### General features of the temperate phage PLg-TB25

**Figure 1:**
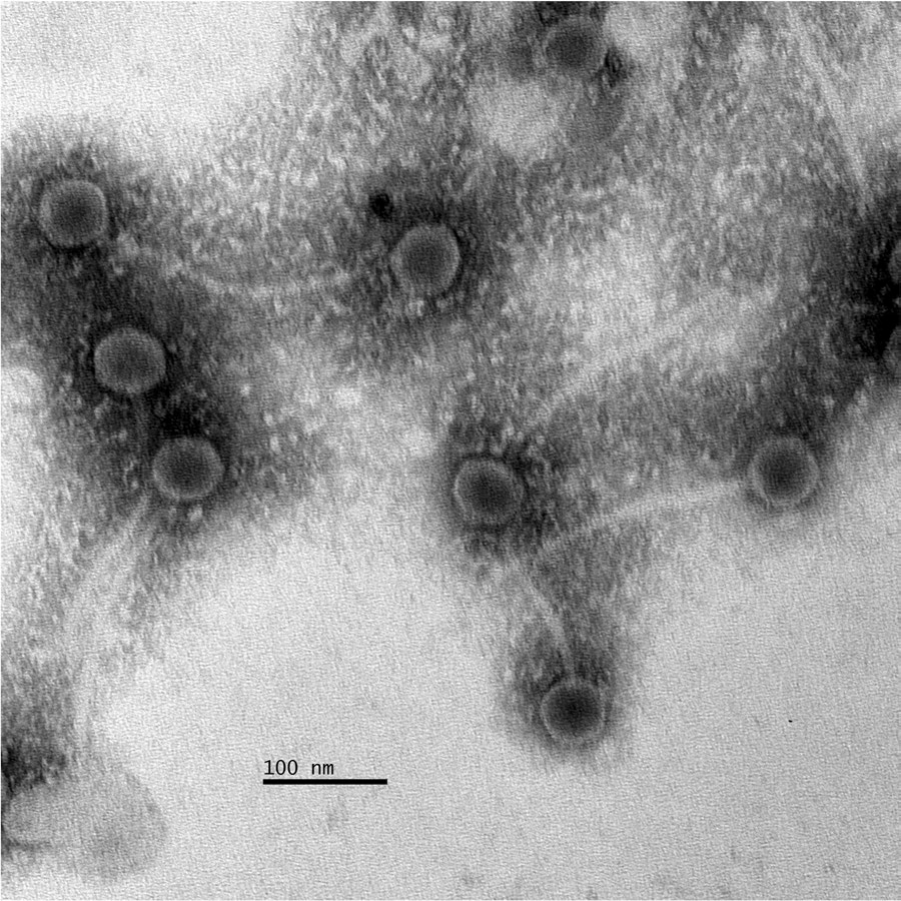
Electron micrograph of the phage PLg-TB25 induced from strain TB25.

*L. garvieae* TB25 was previously isolated from an Italian cheese. A mitomycin C induction assay led to the isolation of an inducible prophage we named PLg-TB25. As shown in Fig. 1, phage PLg-TB25 is characterized by a 60 ± 6 nm icosahedral capsid and a non-contractile tail of 222 ± 6 nm long, 13 ± 3 nm wide, indicating it belongs to the *Siphoviridae* family. It has a double-stranded DNA genome of 38,122 bp and its GC content was calculated to be 34.5%, slightly lower than the GC content (38.1%) of its *L. garvieae* TB25 host^17^. The PLg-TB25 sequence shared no homology with the limited number of known *L*. *garvieae* phages. However, we identified homology with only very short DNA fragments of other *L. lactis* phage genomes. Thus, the *L. garvieae* temperate phage PLg-TB25 is a new member of the *Siphoviridae* family.

### Genome analysis

The search for *orfs* using ORF Finder and RAST Server was limited to those encoding proteins of more than 30 amino acids and flanked by an upstream potential Shine-Dalgarno sequence. The functions of the ORFs were presumed by comparing (BLASTp) deduced protein sequences with the GenBank database as well as by identifying conserved domains. Gene order was started by identifying the gene coding the integrase as the first orf (*orf1*), as done previously for other lactococcal phages^18^. Therefore, the PLg-TB25 genome starts with the divergently oriented lysogenic module (*orf1* to *orf5*), followed by the replication/transcription module (*orf6* to *orf26*), the morphogenesis genes (*orf27* to *orf48*) and finally the lysis module (*orf49* and *orf50*). Similar gene organization has been reported for other lactococcal phages^18^. Of note, while the draft genome of the host *L. garvieae* TB25 strain is available^17^, fragments of the inducible phage genome were found on different contigs of TB25. Yet, the phage gene order was the same on the various bacterial contigs (data not shown) as the gene order obtained in the single assembled contig from the induced phage.

**Figure 2:**
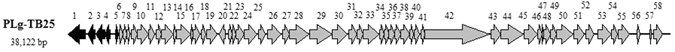
Map of the phage PLg-TB25 genome. Each arrow and number identifies an open reading frame. Black arrows identify the lysogeny module. For specific functions see Table 1.

In total, we identified 58 *orfs* covering 91% of the phage genome (Table 1, Fig. 2). The most common starting codon was AUG (87%), followed by UUG (10%) and GUG (3%). A typical RBS (AGGAGA) preceded only eight *orfs* (*orf5*, *orf10*, *orf11*, *orf21*, *orf30*, *orf34*, *orf38*, and *orf50*). We did not identify any tRNA or recognizable virulence factors in the genome of phage PLg-TB25. Predicted functions were attributed to 31 ORFs (53%). The proteins encoded by the 27 remaining ORFs had no homology with other phage proteins, confirming that phages are sources for novel genes and that the inducible phage PLg-TB25 is new.

**Table 1:** ORFs deduced from the genome of the temperate bacteriophage PLg-TB25.

ORF	Strand^a^	Positions		Size (aa)	MM^b^ kDa	pI	RBS and start codon^c^	Predicted function^d^	Best-match BLASTp result^e^	aa shared with best match/total aa in best match (% ID)	E value	Size^f^(aa)	Accession numbers
		Start	Stop										
1	—	1326	187	379	44	9.4	AGGAGtagaaatcaaATG	Integrase	*L. garvieae*	375/379 (99%)	0.0	379	WP_019292401
2	—	1924	1451	157	18	9.2	AGGAGtattttATG	SHOCT domain	ORF2, *Lactococcus* phage TPW22	81/204 (40%)	4.0E-33	205	AF066865_5
3	—	2540	1950	196	23	4.9	cGGAGgctctATG	—	*L. garvieae*	174/196 (89%)	4.0E-124	196	WP_040086243
4	—	2896	2552	114	13	4.9	gaaAGGttgatactcATG	Transcription regulator	*L. garvieae*	102/114 (89%)	2.0E-67	114	WP_004259391
5	+	3196	3414	72	8	9.3	AGGAGAttATG	—	*L. garvieae*	71/72 (99%)	3.0E-42	72	WP_019335571
6	+	3430	3690	86	11	9.2	AGGAGtaaaaaATG	Excisionase	*L. garvieae*	85/86 (99%)	5.0E-55	86	WP_019292837
7	+	3700	3918	72	9	6.6	AGGAGttaaaATG	—	*L. garvieae*	61/72 (85%)	3.0E-37	72	WP_019299714
8	+	3941	4123	60	7	8.8	AGGAaAtaaaaATG	Transcription regulator	ORF1961, *L. garvieae* DCC43	46/57 (81%)	3.0E-24	58	EKF50671
9	+	4205	4603	132	15	7.8	tGGAGAaataaaaaATG	—	*L. garvieae*	131/132 (99%)	4.0E-88	132	WP_017370187
10	+	4615	5295	226	26	6.8	AGGAGAataatttATG	Topoisomerase	*L. garvieae*	218/226 (96%)	4.0E-156	226	WP_017370188
11	+	5285	5701	138	16	5.2	AGGAGAaagaggaaataaATG	SSB	*L. garvieae*	135/138 (98%)	4.0E-95	138	WP_019299071
12	+	5800	6126	108	13	9.7	tGGAGgaatagATG	HNH endonuclease	*L. garvieae*	106/108 (98%)	6.0E-72	108	WP_017369953
13	+	6126	6887	253	29	7.7	AGGtGgtctaactaATG	DNA replication	*L. garvieae*	212/243 (87%)	8.0E-153	259	WP_017370084
14	+	6896	7060	54	6	9.2	AGGtGcttATG	—					
15	+	7062	7973	303	34	8.4	AGGttAttgatATG	Primosomal protein	Prepilin peptidase, *L. garvieae*	139/301 (46%)	4.0E-80	297	WP_042217561
16	+	7984	8133	49	6	6.6	AGGtGAaaaATG	—	ORF530, *L. garvieae*	45/49 (92%)	2.0E-22	49	CEF50680
17	+	8130	8534	134	15	9.6	gcGAGActtggaaaATG	Resolvase	*RusA*, *L. garvieae*	129/134 (96%)	3.0E-89	134	WP_019293279
18	+	8640	8924	94	11	6.4	AGGAaggggaaaaATG	—	*L. garvieae*	86/93 (92%)	2.0E-54	93	WP_035002155
19	+	8968	9648	226	26	4.7	tGGAGAaacaacATG	5′-deoxyadenosine	*L*. *lactis*	213/226 (94%)	3.0E-157	226	WP_003132900
20	—	10180	9791	129	15	9.2	AGGtaAatATG	Membrane prot.	*L. garvieae*	121/129 (94%)	2.0E-83	129	WP_019293277
21	+	10373	10579	68	7	4.8	AGGAGAataaaacATG	—	*L. lactis*	52/68 (76%)	5.0E-25	68	WP_012897654
22	+	10585	10818	77	9	10.6	tGGAGAataagtcaaATG	—	*L*. *garvieae*	32/52 (62%)	2.0E-10	73	WP_017370067
23	+	10815	11363	182	21	8.9	AGGttAaacaATG	Membrane prot.	*LemA* family protein, *L. lactis*	136/182 (75%)	3.0E-96	184	WP_046780940
24	+	11372	12181	269	31	8.8	AGGtGcaaATG	Membrane prot.	*L. garvieae*	263/269 (98%)	0.0	269	WP_017369938
25	+	12261	12683	140	16	6.6	AGGgGggaaagttTTG	—	*L. garvieae*	136/140 (97%)	5.0E-95	140	WP_017370065
26	+	12856	13686	276	32	5.2	AGGAGtgtattTTG	—	ORF27, phage Tuc2009	178/276 (64%)	5.0E-132	276	NP_108706
27	+	13773	14225	150	17	9.1	AGGtGAgcgattgaGTG	Terminase	*Staphylococcus saprophyticus*	81/150 (54%)	3.0E-39	174	WP_041080371
28	+	14222	15469	415	47	6.2	tGGAGAaattgaaATG	Terminase	*Macrococcus caseolyticus*	212/392 (54%)	2.0E-146	416	WP_012656828
29	+	15484	16941	485	56	5.0	taGAGAgggtgaggataTTG	Portal protein	ORF6, *E. faecium*	204/482 (42%)	8.0E-114	499	WP_047937716
30	+	16928	17902	324	38	9.1	AGGAGAtgtagctcATG	Capsid morphogenesis	*E. dispar*	132/299 (44%)	2.0E-72	296	WP_016173631
31	+	17986	18495	169	19	4.4	AGGAGgggcaaatATG	—					
32	+	18498	18890	130	14	5.0	AGGAGcataaatATG	—	*E. faecalis*	57/106 (54%)	6.0E-31	113	WP_002407384
33	+	18890	19894	334	37	5.2	AGGAacaaaataATG	Major capsid protein	*E. faecium*	132/331 (40%)	1.0E-83	335	WP_002311457
34	+	19915	20229	104	12	4.7	AGGAGAggtgcaaGTG	Head-tail connector	ORF6, *Fructobacillus*	43/96 (45%)	1.0E-22	109	GAO99837
35	+	20230	20535	101	11	8.8	tgggggtattagATG	—	*Staph. pasteuri*	30/96 (31%)	2.0E-06	100	WP_023373491
36	+	20528	20869	113	13	5.2	AGGtAGtggtcATG	Tail-component	ORF10, *L. johnsonii*	49/114 (43%)	7.0E-21	116	EEJ59343
37	+	20869	21255	128	15	4.5	AGGctttttaaataATG	—	*E. faecalis*	35/112 (31%)	2.0E-11	130	WP_016619128
38	+	21267	21845	192	21	5.0	AGGAGAaaaaaaATG	Major tail protein	*Fruct. tropaeoli*	68/184 (37%)	2.0E-30	188	GAP04943
39	+	21863	22099	78	8	4.6	AGGtaAcagaaaaaATG	—	*L. lactis*	37/70 (53%)	3.0E-11	70	WP_023189578
40	+	22114	22461	115	13	5.0	AGGgtAaatcATG	Tail assembly	*E. faecalis*	42/116 (36%)	2.0E-07	132	WP_002363376
41	+	22536	22820	94	11	4.9	AGaAattgaccgcATG	Glycohydrolase					
42	+	22820	26896	1358	143	9.0	AGGAGgcataATG	Tape measure	*E. faecalis*	441/1369 (32%)	0.0	1348	WP_042888997
43	+	26989	27612	207	24	4.9	AGGAaAagTTG	—	*Fruct. fructosus*	68/210 (32%)	3.0E-26	239	WP_010691880
44	+	27612	29060	482	55	5.5	AGGtaAtgatgtaATG	Tail endopeptidase	*Fruct. fructosus*	186/439 (42%)	1.0E-113	612	WP_010691878
45	+	29073	29957	294	34	4.5	AGGgagttacATG	—	*E. faecalis*	79/188 (42%)	3.0E-38	243	ETU52076
46	+	29959	30210	83	9	4.7	AGGgGAattaatATG	—	*E. faecium*	30/78 (38%)	7.0E-12	110	WP_005874742
47	+	30213	30329	38	4	4.7	AGGgGAaataatcATG	—					
48	+	30329	30751	140	15	5.0	AGaAGAagggtggttcaactaATG	—	*L. garvieae*	87/140 (62%)	2.0E-49	133	WP_019292915
49	+	30768	31130	120	14	5.1	AGGAaAaataaaaaTTG	Holin	Holin, *L. garvieae*	118/120 (98%)	4.0E-77	120	WP_019293253
50	+	31114	32181	355	39	5.4	AGGAGAtgaaaATG	Endolysin	1,4-beta-N-acetylmuramidase	346/355 (97%)	0.0	355	WP_019292912
51	+	32252	32914	220	26	5.8	tGGAGActaacaATG	Glucose-1-dehydrogenase	*L. lactis*	68/216 (31%)	2.0E-31	223	WP_023163727
52	+	33010	33525	171	20	5.0	AGGtGcttagaATG	Helix-hairpin	*L. lactis*	69/180 (38%)	4.0E-21	180	WP_046781535
53	+	33779	34609	276	31	4.8	AGGAGctattATG	Nucleoside triphosphate hydrolase	*P-Loop, L. lactis*	187/274 (68%)	4.0E-128	279	WP_003132960
54	+	34611	35018	135	16	8.8	AGGAGgtgtgatATG	—	*L. lactis*	74/136 (54%)	3.0E-34	146	WP_003132961
55	+	35021	35776	251	30	4.9	AGGAGttaaaaTTG	—	*L. lactis*	163/248 (66%)	2.0E-111	248	WP_003132962
56	+	36214	36414	66	7	5.1	AGGgaAatatatatactATG	Cold-shock protein	*L. garvieae*	65/66 (98%)	2.0E-38	66	WP_017369912
57	+	37031	37153	40	5	10	AGGAtAtgatATG	—	ORF1091, *L. garvieae* 49156	39/40 (98%)	9.0E-18	40	BAK58604
58	+	37267	37863	198	23	5.5	AGGAGctagtgATG	Histidine phosphatase	*L. garvieae*	196/198 (99%)	4.0E-144	198	WP_019293168

^a^Orientation of the gene in the genome. ^b^MM, molecular mass. ^c^RBS, ribosomal binding site: uppercase letters represent the hypothetical RBS sequences, bold letters the starting codons. ^d^Indicates no significant matches. ^e^BLASTp result corresponds to second best alignment. ^f^Total size of the aligned proteins.

Within the lysogeny and replication modules, the majority of the ORFs best matched proteins found in strains of *L. garvieae* and *L. lactis*. Conversely, the deduced ORFs involved in the phage’s morphological structure are similar to proteins found in other Gram positive bacteria, such as *Staphylococcus* spp., *Enterococcus* spp., and *Fructobacillus* spp., although with low amino acid identity (31–54%). A 6-kb region containing 8 genes with low GC content (31.5%) was located downstream of the lysis module. One of the genes seemed to code for a cold shock protein^19^. While the function of cold-shock proteins is not fully understood, they often bind nucleic acids and may provide a mechanism for coping with stress and adapting to changing environmental conditions. This additional region at the end of the genome was likely acquired through recombination events or imprecise excision of the prophage.

A comparison between the genomes of phage PLg-TB25 and the PLgT-1 temperate phage from a *L. garvieae* marine fish isolate, revealed similar length (38 kb for PLg-TB25 and 40 kb for PLgT-1) and GC content (35.4% for the marine isolate and 34.5% for the dairy isolate). The 66 *orfs* found in PLgT-1 are organized in modules similar to PLg-TB25 but the gene/protein content is completely different.

### Search for temperate phages in *L. garvieae* genomes

The search for prophages was extended to 16 *L. garvieae* genomes available in GenBank (Supplementary Table S1). As reported in Table 2, eight seemingly complete prophages were found in the genomes of seven *L. garvieae* strains isolated from dairy and fish environments. The genome sizes ranged from 30 to 40 kb and, when possible, the integration site (*att* core) was also determined. Six prophages had lower GC content (34.1–35.9%) compared to the rest of the bacterial genome (37–38%).

**Table 2:** Position, orientation, length, *att* core sequence, %GC and tRNA of the temperate phages in different *L. garvieae* genomes.

Host (source of isolation)	Research procedure	Temperate phage(s)		Contig accession number	Extremities	Length (bp)	*Att* core sequence	%GC	tRNA
TB25 (Italian cheese)	*Ex novo* sequencing	1		KX833905	1–38,122	38,122	—^a^	34.5	—^a^
IPLA 31405 (cow milk)	*Silico*	2	a	NZ_AKFO01000017.1	204,469–239,454	34,986	AACTCCCCTCGCCTCCATTG^b^	36.4	—^a^
			b	NZ_AKFO01000017.1	509,217–478,639	30,579	TTGTGCCAAATTTGTGCCAAA^b^	35.1	—^a^
NBRC 100934 (cow mastitis)	*Silico*	1		NZ_BBJW01000003.1	48,776–12,512	36,265	ATGGGTGGCATGATGTA^b^	37.5	1 (Lys)
ATCC 49156 (diseased yellowtail)	*Silico*	1		NC_015930	1,146,793–1,106,521	40,273	AACTCCCCTCGCCTCCATTGTAT^b^	35.4	2 (Lys, Met)
Lg2 (diseased yellowtail)^c^	*Silico*	1		NC_017490	1,160,852–1,120,580	40,273	AACTCCCCTCGCCTCCATTGTAT^b^	35.4	2 (Lys, Met)
UNIUD 074 (diseased rainbow trout)	*Silico*	1		NZ_AFHF01000007	40,669–2,192	38,478	—^d^	35.9	2 (Ser, Met)
8831 (diseased rainbow trout)	*Silico*	1		NZ_AFCD01000019.1	31,965–251	31,715	—^d^	34.6	1 (Arg)
PAQ102015-99 (rainbow trout)^e^	*Silico*	1		LXWL01000001.1	719,999–756,130	36,132	TCTACTATTGACGTTTAATAATTTAAAAACCCTTGTAAAT	34.1	1 (Arg)

^a^Not found. ^b^ *Att* core has been determined by searching for perfect direct repeats in the vicinity of the phage genome. ^c^ATCC 49156 and Lg2 genomes are co-linears (99% sequence identity). ^d^None sequence more than 10 bp were found. ^e^8831 and PAQ102015-99 genomes are 98% symmetric identity (NCBI data). Lys: lysine, Met: methionine, Ser: serine, Arg: arginine.

**Figure 3:**
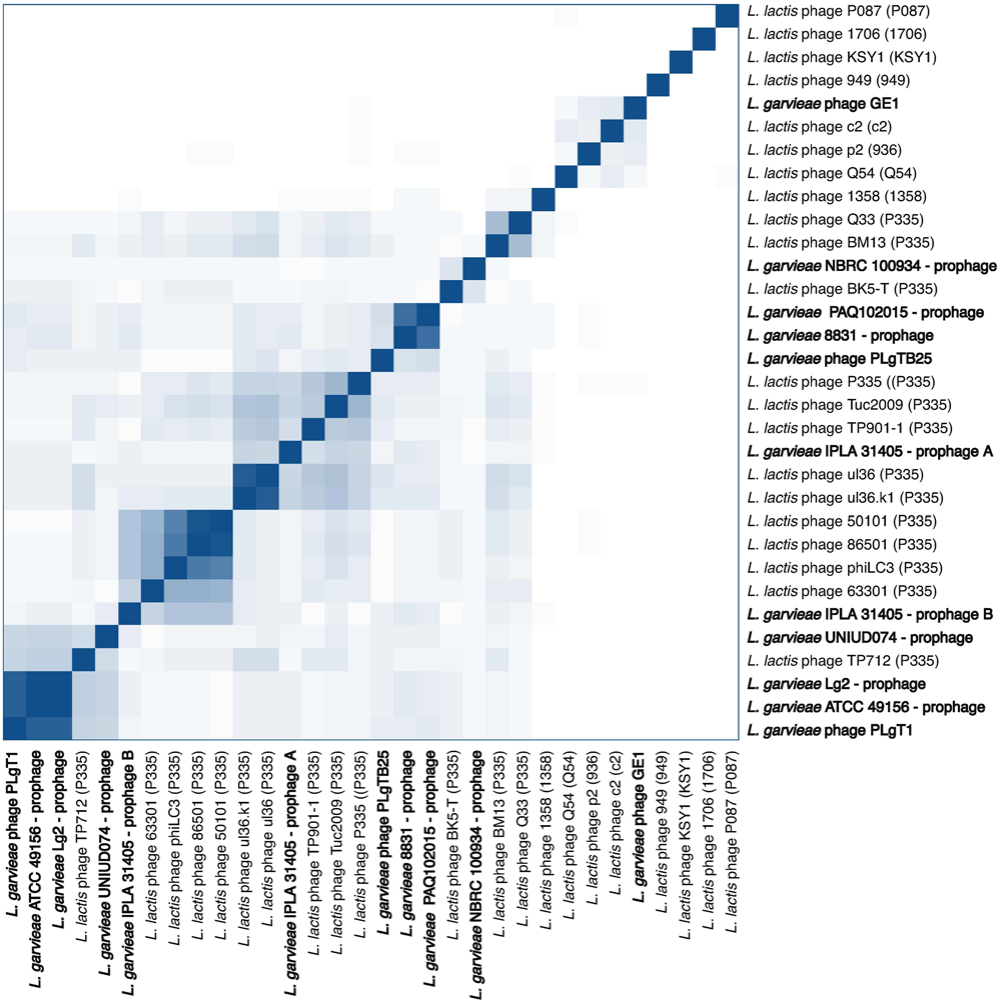
Similarity matrix of 32 lactococcal phages and prophages based on the presence/absence of genes. The heatmap is generated based on the number of proteins shared by phages. Deeper shade of blue indicates a closer relationship.

To verify whether *L*. *garvieae* strains colonizing a similar ecological niche carried similar prophages, we compared the genome of inducible prophage PLg-TB25 with prophages found in the genomes of the two *L*. *garvieae* strains of dairy origin, IPLA 31405 and NBRC 100934. Very low sequence identity was found between these prophages. Moreover, the prophage from NBRC 100934 (PLg-100934) shared low nucleotide identity with other phage genomes available in GenBank. In fact, the closest (with 11% identity) phage genome was the *L*. *lactis* temperate phage BK5-T (P335 group, Fig. 3)^20^.

The genome of PLg-100934 was 36,265 bp in length with a GC content of 37.5%, a value close to its host (38.5%) (Supplementary Table S2). A total of 54 *orfs* were detected, covering 90% of the genome. The majority of the ORFs use AUG as the starting codon (85%), followed by UUG (11%) and GUG (4%). A RBS (AGGAGA) was found upstream of 11 *orfs* (*orf4*, *orf*11, *orf*18, *orf*19, *orf*21, *orf*28, *orf*32, *orf*37, *orf*45, *orf*48, and *orf*52). Genome analysis identified one tRNA (Lys) and no recognizable virulence factors. The genome of PLg-100934 was also divided into four modules: lysogeny (*orf1* to *orf6*), replication/transcription (*orf7* to *orf31*), morphogenesis (*orf32* to *orf50*), and lysis (*orf51* and *orf52*). Predicted functions were attributed to 23 of the 54 *orfs* (42%), including *orf31*, which was predicted to be related to a *L. lactis* homing endonuclease thought to be involved in horizontal gene transfer^21, 22^. As reported for phage PLg-TB25, the PLg-100934 genome carries two extra genes with low GC content (31.8%) downstream of the lysis module. The function of the deduced proteins is unknown.

**Figure 4:**
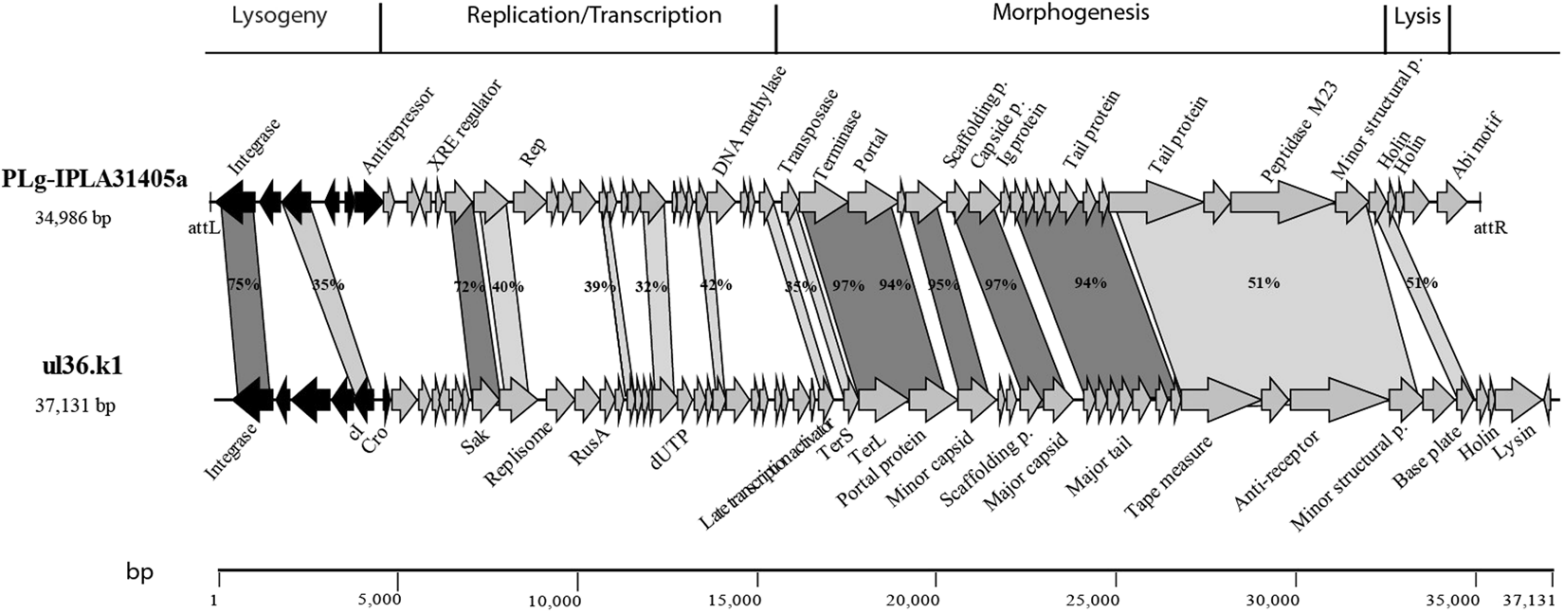
Genomic comparison between *L. garvieae* phage IPLA31405b and *L. lactis* phage ul36.k1. Color shading was used to discriminate between ≥70% amino acid identity (dark color) and ≤69% amino acid identity (light color). The absence of shading indicates no significant similarity. The percent of amino acid identity inside the shading is representative of the aligned region only. Black arrows identify the lysogeny module.

Two prophages were found in the genome of dairy strain *L. garvieae* IPLA 31405^23^, having homology to *L. lactis* phages. The genome of PLg-IPLA31405a was 34,986 bp in length with a GC content of 36.4%. A total of 53 *orfs* were detected, covering 90% of the genome. The genome of the second prophage, PLg-IPLA31405b, was 30,579 bp in length with a GC content of 35.1% and 46 *orfs* covering 85% of the genome. One of the prophages, PLg-IPLA31405a, had >90% nucleotide homology with the virulent *L. lactis* phage ul36^18^ and its mutant ul36.k1 (Fig. 3), the latter being resistant to the AbiK abortive infection mechanism^24^. Half of the deduced ORFs (26/53) had between 32 and 97% amino acid identity to proteins from these *L. lactis* phages (Fig. 4). The morphogenesis module was particularly conserved, suggesting the same morphological features. Both *L. lactis* phages (ul36 and ul36.k1) are virulent members of the P335 group, which contains both temperate and lytic phages^4^. The gene coding for a dUTPase proposed to be used to detect P335 phages was not found in the PLg-IPLA31405a genome^18^.

Interestingly, the deduced protein of a gene found after an *orf* coding for a putative XRE regulator in the PLg-IPLA3145a genome had 72% amino acid identity with the Sak protein of *L. lactis* phages ul36.k1^24^ and ul36.1^25^. Sak is involved in sensitivity/insensitivity to the lactococcal AbiK abortive infection system (Fig. 4). Surprisingly, a gene (*orf53*) coding for a protein sharing a conserved domain with the lactococcal abortive infection system, AbiF (COG4823)^26^, was detected downstream of the lysis module^27^. A phylogenetic analysis was performed using the amino acid sequences of ORF53 (AbiF conserved domain), phage PLg-IPLA 3405a and 20 Abi systems from *L. lactis* ^27–29^. The proteomic phylogenetic tree constructed using MEGA5 software and the neighbour-joining method revealed that the *L. garvieae* Abi-like protein was grouped with other lactococcal Abi systems tested, but diverged in a separate branch (Supplementary Fig. S1).

**Figure 5:**
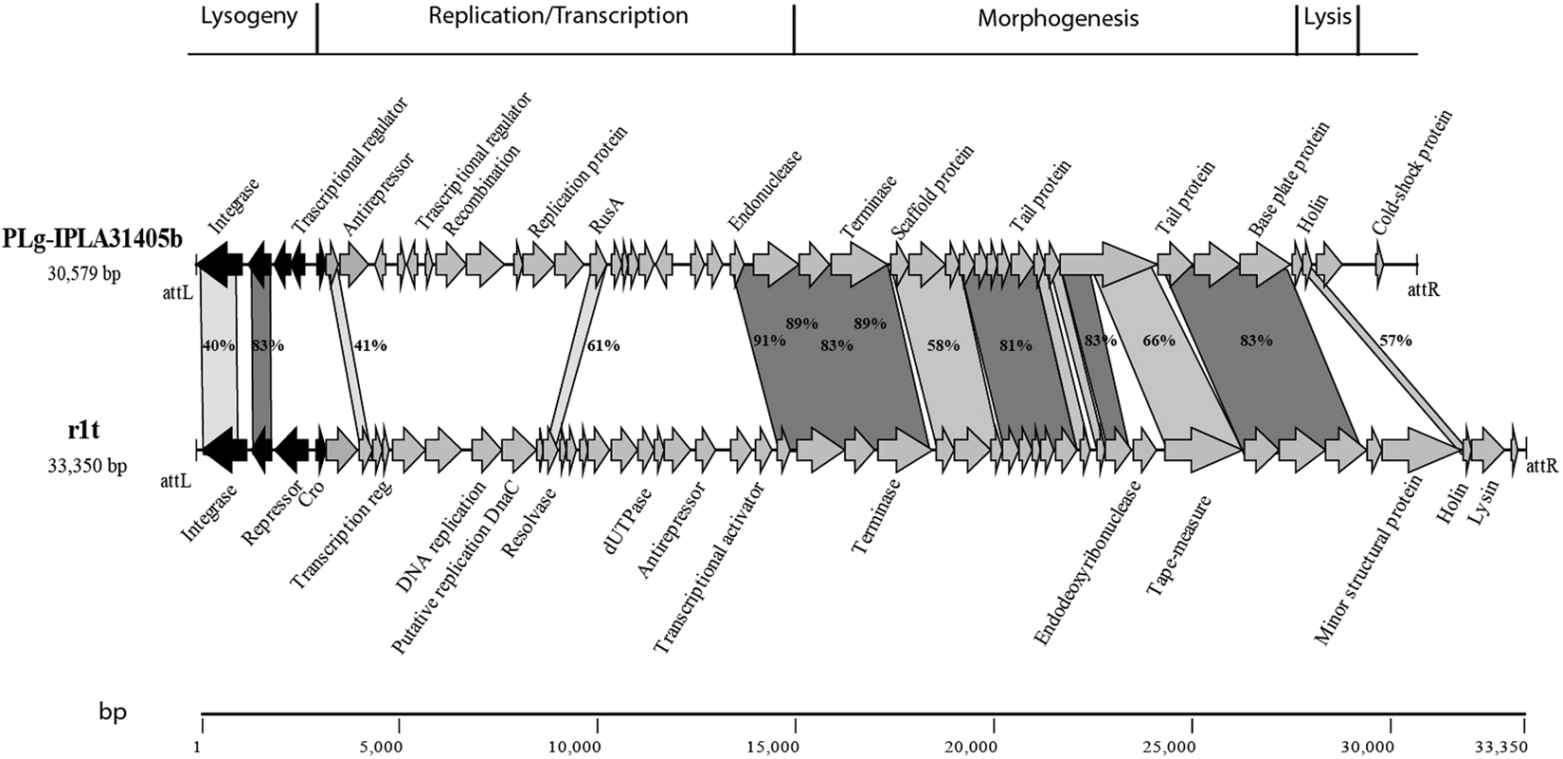
Genomic comparison between *L. garvieae* phage IPLA31405b and *L. lactis* phage r1t. Color shading was used to discriminate between ≥70% amino acid identity (dark color) and ≤69% amino acid identity (light color). The absence of shading means no significant similarity. The percent of amino acid identity inside the shading is representative of the aligned region only. Black arrows identify the lysogeny module.

The other *L. garvieae* IPLA 31405 prophage, PLg-IPLA 31405b, was related to the temperate phage r1t from *L. lactis* ^30^ (Figs 3 and 5). Phage r1t also belongs to the P335 group (subgroup III)^31^. The highest amino acid identity was found with proteins involved in the morphogenesis module (75%). While a gene coding for a dUTPase was not found, an additional gene, located 700 bp downstream from the lysis module, appeared to code for a protein with a conserved cold-shock DNA-binding domain (pfam00313).

Similar comparative genome analyses were performed with prophages harboured by *L. garvieae* strains isolated from fishes, such as ATCC 49156, Lg2 and UNIUD 074 (Table 2). The prophages from *L. garvieae* ATCC 49156 and Lg2 are closely related (99% nucleotide identity) and have significant nucleotide identity (95% over 41% of the genome) with the prophage found in *L. garvieae* strain UNIUD 074 (Fig. 3). Interestingly, they are all related to the temperate phage ɸTP712 found in the widely used plasmid-free laboratory strain *L. lactis* MG1363 and derived from the dairy *L. lactis* strain NCDO 712^32, 33^. Phage ɸTP712 is also related to the sequenced temperate genome, PLgT-1, isolated from a marine environment (Fig. 3). These genomes have a similar size and genome organization. The morphogenesis module is the most conserved region and while we cannot confirm at this time that they are inducible and functional, it is tempting to speculate that at some point they had the same morphological features.

Finally, *L. garvieae* strains 8831 and PAQ102015-99, both isolated from rainbow trout, may have an identical prophage. Due to the genome status (contigs) of strain 8831 we were unable to find the complete phage genome sequence delimited by the *att* sites of PLg-PAQ102015-99 (Table 2). Still, both prophages do not have any significant identity with other known phage genomes but their organization was similar to those discussed above (Supplementary Table S3). Most ORFs seemingly involved in replication and transcription have various levels of similarity with the host proteins of *L. garvieae*. However, the morphogenesis cluster presents the highest nucleotide variability. Seven deduced *orfs* (*orf23, orf24, orf26* to *orf29*, *orf38*) matched (with an amino acid identity ranging from 53 to 82%) proteins found in three species of the genus *Weissella* (*hellenica*, *oryzae* and *koreensis*)^34^. Moreover, seven *orfs* (*orf* 25, *orf30* to *orf34*, *orf37*) displayed similarities with deduced proteins from strains of *Enterococcus gilvus* and *E. faecalis* ^35, 36^. As noted above, a putative homing endonuclease (*orf11*) and a 4.2 kb DNA fragment with lower GC content (31%) were located downstream of the lysis module. Comparison of *L. garvieae* phages with members of the currently recognized 10 * L. lactis* phage groups^4^ revealed that while GE1 is more closely related with phage Q54 (Q54 species) and c2 (c2 species), *L. garvieae* prophages are more related to *L. lactis* phages of the P335 group.

Overall, our comparison of prophages from *L. garvieae* strains isolated from dairy and fish samples indicated low nucleotide identity, highlighting the diversity of lactococcal phages, particularly *L. garvieae* prophages.

## Discussion

The recent isolation of a lytic phage infecting a strain of *L. garvieae* with significant similarities to dairy *L. lactis* phages belonging to the c2 and Q54 groups^14^, raised the question of whether the same was true for temperate *L. garvieae* phages and prophages. Moreover, since little data is available on MGEs that contribute to the evolution and adaptability of the *L. garvieae* species, we characterized an inducible temperate phage and analysed several prophages found within the genomes of *L. garvieae* strains available in public databases. Phage genome sequencing has revealed the presence of several novel genes with unknown functions. While these genes provide limited information on the biology of these phages, their analysis can shed light on their origin and provide underlying information on phage-bacteria interactions.

*L. garvieae* strain TB25 was previously isolated from an Italian cheese sample and was found to possess an inducible prophage belonging to the *Siphoviridae* family. Comparative analyses of the genome of phage PLg-TB25 with the genome of the recently described temperate phage PLgT-1 from a fish *L. garvieae* isolate indicated low nucleotide identity. However, the genome of PLg-TB25 had similar features (genome size, gene organization and GC content) to those observed in other *L. lactis* temperate phages^18^. Yet, the overall low nucleotide identity of phage PLg-TB25 with other phage genomes available in public databases confirmed that it represents a newly functional lactococcal phage. Of note, the inducible phage PLg-TB25 did not infect a panel of 56 strains of *L. lactis* (data not shown).

The analysis of 16 sequenced *L. garvieae* genomes revealed at least three other novel prophage groups. Within the different genomic modules, several genes encode for putative proteins with similarities to deduced proteins from phylogenetically distant genera, such as *Lactobacillus, Weissella*, and *Enterococcus*. In all likelihood, these novel phages are the result of genetic recombination events that have taken place in an environment containing multiple bacterial genera and species, and that have led to subsequent adaptation to a *L. garvieae* host.

The other prophages found in the genome of *L. garvieae* strains show similarity with temperate phages of *L. lactis*, belonging to the P335 group. *L. lactis* phages are currently classified into 10 groups based on genome analysis and phage morphology^4^ but only one group (P335) appears to contain virulent and temperate phages. Some authors have proposed to divide the diverse P335 phage group into subgroups^37^. These observations suggest an evolutionary history in an environment where these two lactococcal bacterial species can thrive, perhaps the dairy ecosystem. Since the GC content of these *L. garvieae* (pro)phages is lower as compared to the GC of their hosts and, in fact, much closer to the GC content of *L. lactis* strains and phages, it is tempting to speculate that they originated from *L. lactis*, while on-going adaptation to a *L. garvieae* host. The analysis of four phage genomes harboured by dairy *L. garvieae* strains also revealed the presence of seemingly additional genes after the lysis module. These genes encode for putative proteins involved in responses to environmental stresses or host strains (cold-shock proteins and defense mechanisms).

Since the *L. garvieae* temperate phage PLgT-1 was previously described to be capable of transduction, thereby possibly playing a role in the genetic evolution and diversification of *L. garvieae* marine strains^16^, it is conceivable to suggest the involvement of the prophages characterized in this study in spreading genes which might contribute to the adaptation of *L. garvieae* to the dairy environment. Mobile Genetic Elements found in strain IPLA 31405 have already been proposed to play an important role in adaptation in milk, through dissemination of the gene for lactose utilization^38^.

Perhaps of interest, no known virulence factors were found in the prophages characterized in this study, even if some of the strains were isolated from infected fishes. While it remains unclear if these *L. garvieae* strains were directly responsible for the reported illnesses, it suggests that the virulence factors are either elsewhere in the bacterial genomes or that new molecules contributing to the pathogenicity of this organism have yet to be discovered.

In conclusion, this study highlights the diversity of *L. garvieae* phages and, in particular, its prophages. While most of our current knowledge about lactococcal phages is derived from the characterization of phages infecting *L. lactis* strains in the cheese and fermented milk industries^39–41^, it appears that the *Lactococcus* phage population is more diverse than previously estimated. In fact, it is plausible that some *L. garvieae* phages might have originated from *L. lactis* while others are the results of recombination events with phages infecting other bacterial genera.

## Methods

### Induction assay and morphology studies

*L. garvieae* strain TB25, previously isolated from an Italian cheese^17^, was grown statically at 30 °C in M17 broth (Pronadisa) containing 1% glucose (GM17) to an optical density at 600 nm (OD_600_) of 0.3. Mitomycin C (Sigma) was added to a concentration of 5 µg/ml and the OD_600_ was measured (in quadruplicate) every 30 min for over 5 hours using a BioTek PowerWave XS2 spectrophotometer (BioTek). Typical induction curves observed with the mitomycin C-containing cultures were characterized by an initial increase in OD_600_ followed by a sharp reduction, compared to the control without mitomycin C.

The presence of induced phages was confirmed by transmission electronic microscopy (TEM). Briefly, the phage lysate was filtered through a 0.45 µm syringe filter and 1 ml was centrifuged at 24,000 × *g* for 1 h at 4 °C (Beckman). The supernatant (approximately 800 µl) was gently discarded and the remaining lysate (approximately 200 µl) was washed twice with 800 µl of ammonium acetate (0.1 M, pH 7.5) then centrifuged (1 h at 24,000 × *g* at 4 °C) and discarded. Next, 10 μl of the remaining phage solution (200 μl) was mixed with 10 μl of 2% uranyl-acetate and deposited on a nickel, Formvar-carbon-coated grid (Pelco International). The liquid was removed after 1 min by touching the edge of the grid with blotting paper. Phage morphology was observed at 80 kV using a JEOL1230 transmission electron microscope (Platforme d’Imagerie Moléculaire et Microscopie of the Université Laval). Capsid size, tail length and tail width were determined by measuring at least 10 phage specimens^31^. The phage was named PLg-TB25.

### Phage DNA extraction

DNA of phage PLg-TB25 was isolated as described previously^42^, with the modifications described here. After DNase treatment to remove free DNA in the phage lysate, the DNAse was inactivated at 65 °C for 30 min. To facilitate the release of phage DNA from the capsid, 200 µl of SDS (20% stock solution) was added, along with 20 µl of proteinase K (stock solution: 20 mg/ml), and samples were incubated at 37 °C for 15 min and then at 60 °C for 30 min.

To sequence the genome of phage PLg-TB25, 90 mL of induced lysate was filtered, and polyethylene glycol (8000, 10% final concentration) and NaCl (final concentration of 0.6 M) were added to the lysate. This mixture was centrifuged at 24,000 × *g* (Beckman) for 1 h at 4 °C. The phage pellet was resuspended in 1 ml of phage buffer (10 mM Tris-HCl pH 7.4, 100 mM NaCl, 10 mM MgSO_4_) and treated with SDS/proteinase K as described above. The DNA was purified using an UltraClean^TM^ Microbial DNA Isolation Kit (MO BIO Laboratories, Inc.).

### Phage DNA sequencing and analysis

A PLg-TB25 sequencing library was first prepared with the Nextera XT DNA Sample Prep Kit (Illumina) according to the manufacturer’s instructions. The library was sequenced using a MiSeq system (2 × 250 nt paired-end). De novo assembly was performed with the ABySS v1.5.2 assembler and CLC v7. Open reading frame (ORF) prediction was carried out using ORF Finder (http://www.ncbi.nlm.nih.gov/gorf/gorf.html) and RAST Server^43^. An ORF was considered valid only if the start codon was AUG, UUG or GUG and coded for at least 30 amino acids (aa). The presence of a ribosomal binding site (RBS) similar to the standard Shine-Dalgarno sequence (AGGAGA) was also determined. Functions and domains were attributed by comparison of the translated products with the database using BLASTp^44^. PSI-BLAST and InterProScan at EMBL-EBI (http://www.ebi.ac.uk/) were used to search for more distant homologous proteins and conserved domains, respectively. The ProtParam tool (http://web.expasy.org/protparam/) was used to determine theoretical molecular masses (MM) and isoelectric points (pI) of the deduced phage proteins. Transfer RNA (tRNA) were predicted using the tRNAscan-SE server^45^ and confirmed using the ARAGORN program^46^. Virulence Factor Databases^47^, together with DBETH^48^, were used to search for virulence factors. Online bioinformatics tools were used with the default settings. Prophage and phage genome maps were generated with BioEdit (http://www.mbio.ncsu.edu/bioedit/bioedit.html) and manually edited in Adobe Illustrator.

### *In silico* search for prophages in the genomes of *L. garvieae* strains

The nucleotide sequences of 16 *L. garvieae* genomes in the NCBI database (Supplementary Table S3) were searched for prophages using PHAST with the default parameters^49^. Sequences of at least 30 kb with genes involved in integration, DNA replication and morphogenesis, were suggestive of complete prophages. Homology searches were performed using BLASTn and BLASTp with default parameters^44, 50^.

### Similarity matrix

The similarity matrix was genereated as previously described^51^. Briefly, all proteins of *L. garvieae* and *L. lactis* (pro)phage were grouped into cluster of orthologous genes using COGsoft^52^ requiring an e-value lower than 1e-3 and a protein alignment covering at least 75% of the length of the longest protein. COGsoft output was parsed to generate a presence/absence binary matrix that was used to calculate de distance between each phages according to the Jaccard index (*dist* function in R). The order of the rows and columns was manually adjusted when needed.

### Nucleotide sequence accession number

The complete annotated genomic sequence of temperate PLg-TB25 phage from *L. garvieae* strain TB25 was deposited in GenBank under accession number KX833905.

## Electronic supplementary material

Supplemental Material

## References

[1] Davies, E. V., Winstanley, C., Fothergill, J. L. & James, C. E. The role of temperate bacteriophages in bacterial infection. *FEMS Microbiol. Lett*. **363**(5), fnw015, doi:10.1093/femsle/fnw015 (2016). 10.1093/femsle/fnw015 26825679

[2] Shabbir MAB (2016). Bacteria vs. bacteriophages: parallel evolution of immune arsenals. Front. Microbiol..

[3] Bakhshinejad B, Sadeghizadeh M (2014). Bacteriophages and their applications in the diagnosis and treatment of hepatitis B virus infection. World J. Gastroenterol..

[4] Deveau H, Labrie SJ, Chopin M-C, Moineau S (2006). Biodiversity and classification of lactococcal phages. Appl. Environ. Microbiol..

[5] Barrangou R (2007). CRISPR provides acquired resistance against viruses in prokaryotes. Science.

[6] Fortier LC, Sekulovic O (2013). Importance of prophages to evolution and virulence of bacterial pathogens. Virulence.

[7] Hayashi T (2001). Complete genome sequence of enterohemorrhagic *Escherichia coli* O157:H7 and genomic comparison with a laboratory strain K-12. DNA Res..

[8] Waldor MK, Mekalanos JJ (1996). Lysogenic conversion by a filamentous phage encoding cholera toxin. Science.

[9] Vendrell D (2006). *Lactococcus garvieae* in fish: A review. Comp. Immunol. Microbiol. Infect. Dis..

[10] Ferrario C, Ricci G, Borgo F, Rollando A, Fortina MG (2012). Genetic investigation within *Lactococcus garvieae* revealed two genomic lineages. FEMS Microbiol. Lett..

[11] Reguera-Brito M (2016). Genetic analysis of human clinical isolates of *Lactococcus garvieae:* relatedness with isolates from foods. Infect. Genet. Evol..

[12] Ferrario C (2013). *Lactococcus garvieae*: where is it from? A first approach to explore the evolutionary history of this emerging pathogen. PLoS One.

[13] Eraclio G, Ricci G, Fortina MG (2015). Insertion sequence elements in *Lactococcus garvieae*. Gene.

[14] Eraclio G (2015). A new virulent phage infecting *Lactococcus* garvieae, with homology to *Lactococcus lactis* phages. Appl. Environ. Microbiol..

[15] Ghasemi SM, Bouzari M, Yoon BH, Chang H-I (2014). Comparative genomic analysis of *Lactococcus garvieae* phage WP-2, a new member of *Picovirinae* subfamily of *Podoviridae*. Gene.

[16] Hoai TD, Nishiki I, Yoshida T (2016). Properties and genomic analysis of *Lactococcus garvieae* lysogenic bacteriophage PLgT-1, a new member of *Siphoviridae*, with homology to *Lactococcus lactis* phages. Virus Res..

[17] Ricci G, Ferrario C, Borgo F, Rollando A, Fortina MG (2012). Genome sequences of *Lactococcus garvieae* TB25, isolated from Italian cheese, and *Lactococcus garvieae* LG9, isolated from Italian rainbow trout. J. Bacteriol..

[18] Labrie S, Moineau S (2002). Complete genomic sequence of bacteriophage ul36: demonstration of phage heterogeneity within the P335 quasi-species of lactococcal phages. Virology.

[19] Keto-Timonen R (2016). Cold shock proteins: a minireview with special emphasis on Csp-family of enteropathogenic *Yersinia*. Front. Microbiol.

[20] Lakshmidevi G, Davidson BE, Hillier AJ (1990). Molecular characterization of promoters of the *Lactococcus lactis* subsp. *cremoris* temperate bacteriophage BK5-T and identification of a phage gene implicated in the regulation of promoter activity. Appl. Environ. Microbiol..

[21] Weisberg RA, Gottesman ME, Hendrix RM, Little JW (1999). Family values in the age of genomics: Comparative analyses of temperate bacteriophage HK022. Annu. Rev. Genet..

[22] Belfort M, Bonocora RP (2014). Homing endonucleases: from genetic anomalies to programmable genomic clippers. Methods Mol. Biol..

[23] Flórez AB (2012). Genome sequence of *Lactococcus garvieae* IPLA 31405, a bacteriocin-producing, tetracycline-resistant strain isolated from a raw-milk cheese. J. Bacteriol..

[24] Labrie SJ, Moineau S (2007). Abortive infection mechanisms and prophage sequences significantly influence the genetic makeup of emerging lytic lactococcal phages. J. Bacteriol..

[25] Bouchard JD, Moineau S (2000). Homologous recombination between a lactococcal bacteriophage and the chromosome of its host strain. Virology.

[26] Garvey P, Fitzgerald GF, Hill C (1995). Cloning and DNA sequence analysis of two abortive infection phage resistance determinants from the lactococcal plasmid pNP40. Appl. Environ. Microbiol..

[27] Chopin MC, Chopin A, Bidnenko E (2005). Phage abortive infection in lactococci: variations on a theme. Curr. Opin. Microbiol..

[28] Durmaz E, Klaenhammer TR (2007). Abortive phage resistance mechanism AbiZ speeds the lysis clock to cause premature lysis of phage-infected *Lactococcus lactis*. J. Bacteriol..

[29] Haaber J, Moineau S, Fortier LC, Hammer K (2008). AbiV, a novel antiphage abortive infection mechanism on the chromosome of *Lactococcus lactis* subsp. *cremoris* MG1363. Appl. Environ. Microbiol..

[30] Van Sinderen D (1996). Sequence analysis and molecular characterization of the temperate lactococcal bacteriophage r1t. Mol. Microbiol..

[31] Samson JE, Moineau S (2010). Characterization of *Lactococcus lactis* phage 949 and comparison with other lactococcal phages. Appl. Environ. Microbiol..

[32] Wegmann U, Overweg K, Jeanson S, Gasson M, Shearman C (2012). Molecular characterization and structural instability of the industrially important composite metabolic plasmid pLP712. Microbiology.

[33] Roces C (2013). Lack of the host membrane protease FtsH hinders release of the *Lactococcus lactis* bacteriophage TP712. J. Gen. Virol..

[34] Fusco V (2015). The genus *Weissella*: taxonomy, ecology and biotechnological potential. Front. Microbiol.

[35] Tyrrell GJ (2002). *Enterococcus gilvus* sp. nov. and *Enterococcus pallens* sp. nov. isolate from human clinical specimens. J. Clin. Microbiol..

[36] Zhang C, Du J, Pen Z (2015). Correlation between *Enterococcus faecalis* and persistent intraradicular infection compared with primary intraradicular infection: a systematic review. J. Endod.

[37] Mahony J (2013). Identification of a new P335 subgroup through molecular analysis of lactococcal phages Q33 and BM13. Appl. Environ. Microbiol..

[38] Flórez AB, Mayo B (2015). The plasmid complement of the cheese isolate *Lactococcus garvieae* IPLA 31405 revealed adaptation to the dairy environment. PLoS One.

[39] Garneau JE, Moineau S (2011). Bacteriophages of lactic acid bacteria and their impact on milk fermentations. Microb. Cell Fact..

[40] Samson JE, Moineau S (2013). Bacteriophages in food fermentations: new frontiers in a continuous arms race. Annu. Rev. Food Sci. Technol..

[41] Mahony J, McDonnell B, Casey E, van Sinderen D (2016). Phage-host interactions of cheese-making lactic acid bacteria. Annu. Rev. Food Sci. Technol..

[42] Moineau S, Pandian S, Klaenhammer TR (1994). Evolution of a lytic bacteriophage via DNA acquisition from the *Lactococcus lactis* chromosome. Appl. Environ. Microbiol..

[43] Aziz RK (2008). The RAST Server: rapid annotations using subsystems technology. BMC Genomics.

[44] Altschul SF (1997). Gapped BLAST and PSI-BLAST: a new generation of protein database search programs. Nucleic Acids Res..

[45] Lowe TM, Eddy SR (1997). tRNAscan-SE: a program for improved detection of transfer RNA genes in genomic sequence. Nucleic Acids Res..

[46] Laslett D, Canback B (2004). ARAGORN, a program to detect tRNA genes and tmRNA genes in nucleotide sequences. Nucleic Acids Res.

[47] Chen L, Xiong Z, Sun L, Yang J, Jin Q (2012). VFDB 2012 update: toward the genetic diversity and molecular evolution of bacterial virulence factors. Nucleic Acids Res..

[48] Chakraborty A, Ghosh S, Chowdhary G, Maulik U, Chakrabarti S (2012). DBETH: a database of bacterial exotoxins for human. Nucleic Acids Res..

[49] Zhou Y, Liang Y, Lynch KH, Dennis JJ, Wishart DS (2011). PHAST: a Fast Phage Search Tool. Nucleic Acids Res.

[50] Altschul SF, Gish W, Miller W, Myers EW, Lipman DJ (1990). Basic local alignment search tool. J. Mol. Biol..

[51] Hamdi S (2017). Characterization of two polyvalent phages infecting Enterobacteriaceae. Sci Rep.

[52] Kristensen DM (2010). A low-polynomial algorithm for assembling clusters of orthologous groups from intergenomic symmetric best matches. Bioinformatics.

