# Supplementary material for: Characterization of prophages of Lactococcus garvieae
Source: Sci Rep. 2017 May 12;7:1856. doi: 10.1038/s41598-017-02038-y (PMC5431838; doi:10.1038/s41598-017-02038-y)
Supplement: Supplementary file 1 — Supplemental Material [file 41598_2017_2038_MOESM1_ESM.docx]

**Characterization of prophages of *Lactococcus garvieae***

**Giovanni Eraclio, Maria Grazia Fortina, Simon J. Labrie, Denise M. Tremblay & Sylvain Moineau**

**SUPPLEMENTARY INFORMATION**

**Supplementary Figure S1.** Phylogenetic tree of Abi mechanisms found in different *Lactococcus* species.

**Supplementary Figure S2.** Genetic alignment of the phage GE1, PLgT-1 and PLg-TB25 where each protein is represented by an arrow. When two proteins share more than 30% identity, they are filled with the same color. White arrows means that these proteins do not share 30% identity with the other proteins.

**Supplementary Table 1.**  *L. garvieae* genomes analyzed in this study.

| ***L. garvieae* strain** | **Origin** | **Source** | **Genome status (scaffolds)** | **Accession Number** |
| --- | --- | --- | --- | --- |
| Lg-ilsanpaik-gs201105 | Clinical isolate | Human gall gladder | Draft (53) | NZ_JPUJ01000000 |
| 21881 | Clinical isolate | Human blood | Draft (91) | AFCC00000000.1 |
| ATCC 49156 | Fish | Diseased yellowtail | Complete | NC_015930 |
| LG2 | Fish | Diseased yellowtail | Complete | NC_017490 |
| Lg9 | Fish | Diseased rainbow trout | Draft (140) | AGQY00000000.1 |
| PAQ102015-99 | Fish | Rainbow trout | Draft (14) | NZ_LXWL01000000.1 |
| UNIUD 074 | Fish | Diseased rainbow trout | Draft (25) | AFHF00000000.1 |
| 122061 | Fish | Diseased yellowtail | Complete | AP017373 |
| 8831 | Fish | Diseased rainbow trout | Draft (87) | AFCD00000000.1 |
| I113 | Food | Meat products | Draft (49) | AMFD00000000.1 |
| Tac2 | Food | Turkey meat | Draft (97) | AMFE00000000.1 |
| IPLA 31405 | Milk | Cow milk | Draft (23) | AKFO00000000.1 |
| M14 | Milk | Fermented milk | Draft (13) | NZ_CCXC01000000 |
| NBRC 100934/DSMZ 20684^T^ | Milk | Cow mastitis | Draft (56) | NZ_BBJW00000000.1 |
| DCC43 | Other origins | Mallard duck intestines | Draft (68) | AMQS00000000.1 |
| TRF1 | Other origins | Snake fecal material | Draft (112) | AVFE00000000.1 |

**Supplementary Table 2**.ORFs deduced from the genome of temperate bacteriophage PLg-100934.

| **ORF** | **Strand^a^** | **Position** | | **ORF size (aa)** | **MM^b^ (kDa)** | **pI** | **Putative RBS and start codon^c^** | **Predicted function^d^** | **BLASTp result^e^** | **# aa shared /total # aa in BLASTp match (% ID)** | **E value** | **Size^f^ (aa)** | **Accession numbers** |
| --- | --- | --- | --- | --- | --- | --- | --- | --- | --- | --- | --- | --- | --- |
|  |  | **Start** | **Stop** |  |  |  |  |  |  |  |  |  |  |
| 1 | - | 1251 | 106 | 381 | 4 | 9.6 | AGGAGtagaaatcaa**ATG** | Integrase | Int, prophage L54a of *L. piscium* | 252/381 (66%) | 0.0 | 381 | CEN29374.1 |
| 2 | - | 1560 | 1384 | 58 | 7 | 9.2 | GGAGttcgacta**ATG** | - |  |  |  |  |  |
| 3 | - | 1895 | 1560 | 111 | 13 | 4.6 | AGGAGgcaat**ATG** | - | H.p., *L. lactis* | 56/111 (50%) | 3.00E-34 | 111 | WP_014570766.1 |
| 4 | - | 2345 | 1911 | 144 | 16 | 5.3 | AGGAGAtttt**ATG** | - | ORF955, H.p., *L. lactis* | 81/144 (56%) | 9.00E-44 | 144 | WP_021722776.1 |
| 5 | - | 2702 | 2370 | 110 | 13 | 7.7 | AGGtGcaaaa**ATG** | Transcriptional regulator | cro/CI transcriptional regulator *E. faecalis* | 48/103 (47%) | 2.00E-26 | 107 | WP_010826721.1 |
| 6 | + | 2856 | 2981 | 41 | 5 | 10.1 | AtcAaAtaca**ATG** | - |  |  |  |  |  |
| 7 | + | 3120 | 3359 | 79 | 9 | 9 | AGGAGcatat**ATG** | - | H.p., *L. lactis* | 64/74 (86%) | 1.00E-40 | 76 | WP_058221518.1 |
| 8 | + | 3352 | 3657 | 101 | 12 | 8.9 | AGGtagtcA**ATG** | - | H.p., *L. piscium* | 50/88 (57%) | 7.00E-28 | 101 | WP_047916351.1 |
| 9 | + | 3660 | 4352 | 230 | 26 | 7.8 | AGGAatttataaga**ATG** | Anti-repressor protein | *AntA*, *L*. *garvieae* | 213/233 (91%) | 1.00E-149 | 233 | WP_040086487.1 |
| 10 | + | 4370 | 4486 | 38 | 4 | 6 | AGGgatattc**ATG** | - | ORF464, H.p., *L. garvieae* ATCC 49156 | 32/38 (84%) | 3.00E-12 | 38 | EOT33274.1 |
| 11 | + | 4497 | 4784 | 95 | 11 | 5.9 | AGGAGAaaata**ATG** | - | H.p., *L. garvieae* | 30/92 (33%) | 9.00E-08 | 95 | WP_042753188.1 |
| 12 | + | 4786 | 4986 | 66 | 8 | 9.4 | AGGtaAataat**ATG** | - | H.p., *L. garvieae* | 55/65 (85%) | 3.00E-32 | 73 | WP_003136214.1 |
| 13 | + | 4979 | 5092 | 37 | 4 | 4.6 | AGGAGcacaca**GTG** | - | ORF1262, H.p., *L. lactis* | 30/37 (81%) | 1.00E-10 | 37 | KGF76748.1 |
| 14 | - | 5780 | 5109 | 223 | 25 | 5.3 | atGGAGAaaat**ATG** | - | H.p., *L. lactis* | 143/223 (64%) | 4.00E-100 | 223 | WP_033900168.1 |
| 15 | + | 6009 | 6830 | 273 | 32 | 9.4 | AGGtGAtaga**TTG** | Integrase | Int., *L. garvieae* | 198/273 (73%) | 4.00E-138 | 273 | WP_003136217.1 |
| 16 | + | 7266 | 7409 | 47 | 6 | 9.3 | AGGgtcgtcct**ATG** | Ribbon-helix-helix protein | - |  |  |  |  |
| 17 | + | 7534 | 7914 | 126 | 15 | 8.7 | AGGtGttttc**ATG** | - | H.p., *Lactococcus* spp. | 60/108 (56%) | 3.00E-36 | 109 | WP_061412641.1 |
| 18 | + | 7911 | 8648 | 245 | 28 | 9.3 | AGGAGAagcaat**ATG** | Helix-turn-helix domain | ORF36, H.p., *L. lactis* | 167/245 (68%) | 7.00E-87 | 249 | WP_046782081.1 |
| 19 | + | 8658 | 8822 | 54 | 6 | 8.1 | AGGAGAtttgt**ATG** | - | H.p., *L. lactis* | 23/49 (47%) | 2.00E-05 | 54 | WP_010905352.1 |
| 20 | + | 8822 | 9715 | 297 | 35 | 6.9 | AGGAaAgta**ATG** | Chromosomal replication initiator protein DnaA | Prepilin peptidase, *L. garvieae* | 289/297 (97%) | 0.0 | 297 | WP_003136220.1 |
| 21 | + | 9727 | 10440 | 237 | 28 | 4.9 | AGGAGAaacaag**ATG** | - | ORF1642, H.p., *L.garvieae* | 93/191 (49%) | 6.00E-36 | 258 | WP_003136582.1 |
| 22 | + | 10437 | 10736 | 99 | 12 | 4.6 | AGGtGAaaga**ATG** | - | H.p., *L. lactis* | 48/106 (45%) | 2.00E-11 | 95 | WP_010905950.1 |
| 23 | + | 10733 | 11062 | 109 | 12 | 4.5 | tGGAGgaaga**ATG** | - | H.p., *L.garvieae* | 44/67 (66%) | 1.00E-19 | 132 | WP_019299674.1 |
| 24 | + | 11055 | 11210 | 51 | 6 | 5.2 | AGGgGAag**ATG** | - |  |  |  |  |  |
| 25 | + | 11203 | 11430 | 75 | 9 | 4.7 | AGGgagcggcg**ATG** | - | H.p., *L. lactis* | 69/73 (95%) | 5.00E-39 | 145 | WP_012897650.1 |
| 26 | + | 11457 | 11666 | 69 | 8 | 5.2 | cGGAtgag**TTG** | - | H.p., *L. lactis* | 59/68 (87%) | 7.00E-37 | 80 | GI:752479463 |
| 27 | + | 11760 | 11948 | 62 | 7 | 9.8 | AGGAGtgttggga**ATG** | - |  |  |  |  |  |
| 28 | + | 12053 | 12583 | 176 | 21 | 5.2 | AGGAGAaagaa**ATG** | - | sugar-phospahte nucleotidyltransferase, *C. maltaromaticum* | 118/176 (67%) | 2.00E-84 | 180 | WP_035065602.1 |
| 29 | + | 12580 | 12795 | 71 | 9 | 11.5 | AaGAGgtagtcaa**ATG** | - | H.p.,*L. garvieae* | 55/71 (77%) | 1.00E-28 | 71 | WP_042753341.1 |
| 30 | + | 12982 | 13419 | 145 | 17 | 8.5 | AGGAGgttaaagtt**TTG** | - | ORF1215, H.p., *L. lactis* subsp. *cremoris* | 57/89 (64%) | 5.00E-33 | 138 | KKW72760.1 |
| 31 | + | 13926 | 14426 | 166 | 20 | 9.6 | AGGAaggaatttaaaatt**ATG** | HNH endonuclease | HNH endonuclease *L. lactis* | 106/170 (62%) | 7.00E-71 | 170 | WP_047205846.1 |
| 32 | + | 14547 | 14999 | 150 | 17 | 5.4 | AGGAGAttattt**TTG** | Terminase | Terminase *L. lactis* | 125/147 (85%) | 1.00E-87 | 150 | WP_014573149.1 |
| 33 | + | 14992 | 16905 | 637 | 74 | 5.1 | gGGgGActtcttcg**ATG** | Terminase | ORF31, Terminase large subunit, phage P335 | 525/635 (83%) | 0.0 | 646 | NP_839922.1 |
| 34 | + | 16874 | 17083 | 69 | 8 | 8.1 | AGaAGAttttg**ATG** | - | ORF1056, H.p., *L. lactis* | 55/69 (80%) | 2.00E-28 | 69 | WP_015966929.1 |
| 35 | + | 17086 | 18291 | 401 | 45 | 5.9 | AaGAGAggtgattaatt**TTG** | Phage portal | Phage portal protein *L. lactis* | 282/391(72%) | 0.0 | 390 | WP_023189043.1 |
| 36 | + | 18312 | 19016 | 234 | 26 | 4.6 | AGGAGgtcaagg**ATG** | Protease | Peptidase *L. lactis* | 184/234 (79%) | 5.00E-131 | 234 | WP_032398674.1 |
| 37 | + | 19029 | 20282 | 417 | 46 | 4.9 | AGGAGActcaaa**ATG** | Phage capside | Phage capsid protein *L. lactis* | 313/408 (77%) | 0.0 | 409 | WP_012898017.1 |
| 38 | + | 20293 | 20628 | 111 | 13 | 4.2 | AGGgGtgacag**ATG** | Phage gp6-like head-tail connector protein | ORF2340, DNA packaging *L. plantarum* | 48/106 (45%) | 8.00E-22 | 112 | CDN29021.1 |
| 39 | + | 20621 | 20968 | 115 | 13 | 9.1 | cGGAGAcggtg**ATG** | Phage head-tail joining protein | Head-tail joining protein *L. sakei* | 61/113 (54%) | 2.00E-39 | 115 | WP_035147673.1 |
| 40 | + | 20971 | 21381 | 136 | 16 | 6 | gaaAGggtgact**ATG** | Putative tail-component | ORF10, H.p., *Lactobacillus sakei* | 68/128 (53%) | 9.00E-40 | 136 | WP_035147674.1 |
| 41 | + | 21381 | 21764 | 127 | 15 | 5.5 | AGGtGAgaatta**ATG** | - | ORF806, H.p., *Lactobacillus sakei* | 58/126 (46%) | 2.00E-37 | 128 | WP_035147676.1 |
| 42 | + | 21766 | 22422 | 218 | 23 | 5 | AGGAGcattaaa**ATG** | Phagetail | Tail protein, *Lactobacillus sakei* | 142/215 (66%) | 7.00E-97 | 222 | WP_035147678.1 |
| 43 | + | 22447 | 22860 | 137 | 16 | 4.6 | AGGAacttacgaca**ATG** | - | H.p., *Lactobacillus reuteri* | 45/123 (37%) | 2.00E-16 | 128 | WP_011953494.1 |
| 44 | + | 23067 | 26939 | 1290 | 139 | 9.3 | AGGgGAaaac**ATG** | Phage-related tail protein | phage tail length tape measure protein *Oenococcus oeni* | 531/1275 (42%) | 0.0 | 1218 | WP_032816177.1 |
| 45 | + | 26960 | 27190 | 76 | 9 | 4.7 | AGGAGAagaga**ATG** | - | H.p., *Lactococcus lactis* | 35/69 (51%) | 4.00E-13 | 68 | WP_021214423.1 |
| 46 | + | 27563 | 28399 | 278 | 32 | 5.1 | AGGAGtccca**TTG** | Phage tail protein | H.p., *Lactobacillus sakei* | 154/278 (55%) | 2.00E-107 | 278 | WP_035147684.1 |
| 47 | + | 28392 | 29894 | 500 | 56 | 4.9 | gacctttagtttcccttttctct**ATG** | Prophage endopeptidase tail | H.p., *Lactobacillus sakei* | 238/431 (55%) | 1.00E-173 | 508 | WP_035147685.1 |
| 48 | + | 29891 | 30928 | 345 | 39 | 5.1 | AGGAGAaaac**ATG** | Hydrolase | ORF879, Phage MSP (*E. faecalis* MTUP9) | 264/345 (77%) | 0.0 | 345 | KAJ80425.1 |
| 49 | + | 30939 | 31598 | 219 | 25 | 4.4 | AGGAaAagaat**ATG** | - | Prophage Lp1 protein 54, *E. faecalis* | 170/219 (78%) | 5.00E-122 | 219 | KAJ80426.1 |
| 50 | + | 31612 | 32151 | 179 | 20 | 4.9 | AGGAaAattaca**ATG** | - | *H.p., L. garvieae* | 75/113 (66%) | 2.00E-48 | 303 | WP_014024883.1 |
| 51 | + | 32173 | 32439 | 88 | 10 | 8 | AGGgGgagaaat**ATG** | Haemolysin XhlA | H.p., *Lactobacillus farraginis* | 30/83 (36%) | 5.00E-06 | 88 | WP_035180475.1 |
| 52 | + | 32459 | 33514 | 351 | 38 | 6.4 | AGGAGAattttagaaa**ATG** | Lyzozyme | 1,4-beta-N-acetylmuramidase, *L. garvieae* | 321/355 (90%) | 0.0 | 355 | WP_019299026.1 |
| 53 | + | 34275 | 35279 | 334 | 38 | 5.1 | AGGAtAaa**ATG** | - | H.p., *Lactococcus garvieae* | 229/231 (99%) | 1.00E-161 | 231 | AEK12129.1 |
| 54 | + | 35281 | 35949 | 222 | 26 | 9.5 | AGGtGAtggattaaa**A**GA | - | H.p., *Lactobacillus plantarum* | 101/228 (44%) | 4.00E-46 | 220 | KGH42505.1 |

^a^Orientation of the gene in the genome.

^b^MM, molecular mass.

^c^RBS, ribosomal binding site. The uppercase letter represent the hypothetical RBS sequences and in bold the starting codon, whereas the lowercase letter represent the nucleotides between the RBS sequence and the starting codon and also nucleotide after the RBS.

^d^ – indicates no significant matches.

^e^BLATp result corresponds to second best alignment.

^f^Total size of the aligned protein.

**Supplementary Table 3**. ORFs deduced from the genome of temperate bacteriophage found in PLg-PAQ102015-99.

| **ORF** | **Strand^a^** | **Position** | | **ORF size (aa)** | **MM^b^ (kDa)** | **pI** | **Putative RBS and start codon^c^** | **Predicted function^d^** | **BLASTp result^e^** | **# aa shared /total # aa in BLASTp match (% ID)** | **E value** | **Size^f^ (aa)** | **Accession numbers** |
| --- | --- | --- | --- | --- | --- | --- | --- | --- | --- | --- | --- | --- | --- |
|  |  | **Start** | **Stop** |  |  |  |  |  |  |  |  |  |  |
| 1 | - | 1228 | 104 | 374 | 44 | 9.5 | AGGAGtagaaatcaa**ATG** | Integrase | Int, *L. lactis* | 258/380 (68%) | 0.0 | 380 | WP_021037034.1 |
| 2 | - | 1937 | 1359 | 192 | 20 | 7.8 | AGGAGAaaaatttta**ATG** | Putative membrane protein | H.p., *L*. *garvieae* | 171/192 (89%) | 1.00E-117 | 192 | WP_003134907.1 |
| 3 | - | 2580 | 1990 | 196 | 23 | 5 | AGactAatcgggggctct**ATG** | - | H.p., *L. garvieae* | 190/196 (97%) | 6.00E-136 | 196 | WP_040086243.1 |
| 4 | - | 2945 | 2592 | 117 | 13 | 4.8 | AGGAGAcctatata**ATG** | Transcriptional regulators | XRE transcriptional regulator, *L*. *garvieae* | 115/117 (98%) | 2.00E-75 | 117 | WP_019292841.1 |
| 5 | - | 4033 | 3239 | 264 | 30 | 5.4 | AGGAGAtttt**ATG** | - | H.p., *L.lactis* | 160/263 (61%) | 2.00E-105 | 256 | WP_058211621.1 |
| 6 | + | 4188 | 4418 | 76 | 9 | 7.8 | AGGAtAaaa**ATG** | - | H.p., *L. garvieae* | 68/75 (91%) | 1.00E-42 | 75 | WP_003134901.1 |
| 7 | + | 4431 | 4682 | 83 | 10 | 9.4 | AGGggtaaac**ATG** | - |  |  |  |  |  |
| 8 | + | 4699 | 4848 | 49 | 6 | 7.9 | AGGAGcaact**ATG** | - | ORF1268, H.p., *L. garvieae* | 46/49 (94%) | 1.00E-23 | 49 | CEF51378.1 |
| 9 | + | 5065 | 5841 | 258 | 30 | 5 | AGGAattaaaa**ATG** | Recombination protein | Recombination protein Bet, *L*. *garvieae* | 250/258 (97%) | 0.0 | 258 | WP_004257695.1 |
| 10 | + | 5842 | 6807 | 321 | 38 | 5.1 | AGGActtttaa**ATG** | - | ORF1351, H.p., *L. garvieae* | 293/321 (91%) | 0.0 | 321 | WP_004257691.1 |
| 11 | + | 7040 | 7366 | 108 | 13 | 9.5 | AGGAatat**ATG** | HNH endonuclease | ORF3, Endonuclease *L. garvieae* | 107/108 (99%) | 9.00E-72 | 108 | WP_017369953.1 |
| 12 | + | 7366 | 8151 | 261 | 30 | 8.7 | AGGtGgtccagcta**ATG** | Helix-turn-helix domain | ORF36, HTH, *L. garvieae* | 245/262 (94%) | 2.00E-173 | 261 | WP_026063845.1 |
| 13 | + | 8135 | 8419 | 94 | 11 | 9.4 | AGGAGAtagcag**ATG** | - | H.p., *L. garvieae* | 83/93 (89%) | 6.00E-56 | 93 | WP_017369951.1 |
| 14 | + | 8416 | 8820 | 134 | 15 | 9.5 | gGGtaAgtaagac**ATG** | Resolvase *RusA* | *RusA*, *L. garvieae* | 129/134 (96%) | 2.00E-89 | 134 | WP_019335580.1 |
| 15 | + | 8927 | 9208 | 93 | 11 | 9.3 | AaGAaAggggagaa**ATG** | HD nuclease superfamily | H.p., *L. garvieae* | 74/90 (82%) | 2.00E-45 | 90 | WP_017369948.1 |
| 16 | - | 9702 | 9313 | 129 | 15 | 9 | AGaAagtaggtaaat**ATG** | - | ORF805, H.p., *L. garvieae* | 121/129 (94%) | 2.00E-83 | 129 | WP_019335578.1 |
| 17 | - | 10218 | 9820 | 132 | 16 | 7.8 | AGGAGggccactat**ATG** | Arsenate reductase | *ArsR*, *L*. *garvieae* TRF1 | 126/132 (95%) | 1.00E-87 | 132 | ETD04164.1 |
| 18 | + | 10409 | 10810 | 133 | 16 | 9.2 | AGGAagtttattc**ATG** | - | ORF722, H.p., *L. garvieae* | 132/133 (99%) | 3.00E-90 | 133 | WP_017369937.1 |
| 19 | + | 11108 | 12238 | 376 | 41 | 5.9 | AaGAGgtaaaca**ATG** | Lysozyme-like | H.p., *L*. *garvieae* | 159/180 (88%) | 3.00E-103 | 376 | WP_042218703.1 |
| 20 | + | 12458 | 13090 | 210 | 23 | 4.9 | AGGAGgtaggaa**ATG** | - | H.p., *L. garvieae* TRF1 | 131/138 (95%) | 4.00E-89 | 210 | ETD04990.1 |
| 21 | + | 13375 | 13923 | 182 | 21 | 8.9 | AGGgGAaaat**ATG** | - | H.p., *L. garvieae* | 77/164 (47%) | 6.00E-44 | 162 | WP_042217496.1 |
| 22 | + | 14135 | 14626 | 163 | 18 | 7.8 | AGGAGggaggt**ATG** | Terminase | Terminase, *L*. *garvieae* | 118/150 (79%) | 3.00E-78 | 150 | WP_014024903.1 |
| 23 | + | 14616 | 15899 | 427 | 49 | 7.7 | AGGggttcaaag**ATG** | Terminase | Terminase, *Weissella hellenica* | 290/426 (68%) | 0.0 | 426 | WP_042492710.1 |
| 24 | + | 15901 | 17496 | 531 | 61 | 4.5 | AGGAaAttaat**ATG** | Portal protein | PPP, *Weissella hellenica* | 328/497 (66%) | 0.0 | 496 | WP_042492712.1 |
| 25 | + | 17480 | 18370 | 296 | 34 | 8.5 | AGGAGtcagaag**ATG** | Head morphogenesis | Morphogenesis protein, *E*. *gilvus* | 132/292 (45%) | 2.00E-85 | 296 | WP_010780109.1 |
| 26 | + | 18513 | 19082 | 189 | 21 | 4.7 | AGGAagaatt**ATG** | - | H.p., *Weissella hellenica* | 90/144 (63%) | 3.00E-52 | 168 | WP_042492716.1 |
| 27 | + | 19093 | 19959 | 288 | 32 | 5 | AGGAGAaaaatt**ATG** | - | H.p., *Weissella oryzae* | 236/287 (82%) | 9.00E-176 | 290 | WP_027699892.1 |
| 28 | + | 20037 | 20495 | 152 | 15 | 4.8 | AGGAGggctt**ATG** | Bacterial Ig-like domain | Head protein, *Weissella koreensis* | 81/147 (55%) | 5.00E-37 | 150 | WP_006845687.1 |
| 29 | + | 20508 | 20852 | 114 | 13 | 4.8 | AGGAGgtaatg**ATG** | Phage head-tail connector | H.p., *Weissella oryzae* | 72/110 (65%) | 8.00E-44 | 111 | WP_027699890.1 |
| 30 | + | 20849 | 21124 | 91 | 11 | 8.6 | AGcgaggtttat**ATG** | - | H.p., *Enterococcus faecalis* | 34/92 (37%) | 4.00E-09 | 92 | WP_016624515.1 |
| 31 | + | 21124 | 21474 | 116 | 13 | 9.9 | tGGAGgtgattta**ATG** | Putativetail-component | H.p., *Enterococcus faecalis* | 40/108 (37%) | 4.00E-19 | 112 | WP_010708429.1 |
| 32 | + | 21471 | 21833 | 120 | 14 | 5 | AGGtGgttga**ATG** | Zincpeptidases | H.P., *Enterococcus gilvus* | 61/113 (54%) | 5.00E-37 | 119 | WP_010780116.1 |
| 33 | + | 21844 | 22491 | 215 | 23 | 4.2 | AGGAGAaaat**ATG** | Phage major tail protein | MTP, *Enterococcus faecalis* | 129/191 (68%) | 3.00E-87 | 192 | WP_010708431.1 |
| 34 | + | 22561 | 23004 | 147 | 16 | 5.1 | AGagGAaaaatt**ATG** | - | ORF1794, H.p., *Enterococcus faecalis* | 52/137 (38%) | 1.00E-17 | 135 | WP_010826655.1 |
| 35 | + | 23049 | 23330 | 93 | 11 | 9.8 | AGAtatcaatcttatt**ATG** | - |  |  |  |  |  |
| 36 | + | 23330 | 26029 | 899 | 95 | 9.6 | AGGgaggtaaata**ATG** | Phage protein | Tail protein, *Lactococcus lactis* | 361/883 (41%) | 0.0 | 833 | WP_011676059.1 |
| 37 | + | 26030 | 26875 | 281 | 33 | 5.1 | AGGAatataa**ATG** | Phage tail protein | H.p., *Enterococcus gilvus* | 139/282 (49%) | 4.00E-93 | 282 | WP_010780120.1 |
| 38 | + | 26872 | 28974 | 700 | 78 | 4.7 | AGtgGAgttttactt**ATG** | Endopeptidase tail | H.p., *Weissella oryzae* | 377/717 (53%) | 0.0 | 696 | WP_027699884.1 |
| 39 | + | 28964 | 30307 | 447 | 49 | 4.8 | AGGAaAtatgA**ATG** | - | H.p., *Lactococcus garvieae* | 195/406 (48%) | 2.00E-103 | 401 | WP_019299182.1 |
| 40 | + | 30348 | 30710 | 120 | 14 | 5.2 | AGGAaAaataaaaa**TTG** | - | Holin, *Lactococcus garvieae* | 118/120 (98%) | 4.00E-77 | 120 | WP_019299702.1 |
| 41 | + | 30694 | 31818 | 374 | 41 | 5.3 | AGGAGAtgaaa**ATG** | Lysine motif | Lysin, *Lactococcus garvieae* | 342/374 (91%) | 0.0 | 374 | WP_019291755.1 |
| 42 | + | 31893 | 32579 | 228 | 27 | 5.9 | AGGAGAaatttaa**ATG** | - | H.p., *Lactococcus garvieae* | 213/228(93%) | 3.00E-152 | 228 | WP_019291754.1 |
| 43 | + | 32817 | 33281 | 154 | 18 | 8.7 | AGGAataggta**TTG** | - | H.p., *Lactococcus lactis* | 120/154(78%) | 4.00E-86 | 154 | WP_058211395.1 |
| 44 | + | 33285 | 34091 | 268 | 31 | 5.0 | AGGAcgggctaaaca**ATG** | - | ORF1828, H.p., *Lactococcus lactis* | 232/267(87%) | 1.00E-171 | 267 | WP_058211396.1 |
| 45 | + | 34999 | 35121 | 40 | 5 | 10 | AGGAtAtggt**ATG** | - | ORF1091, H.p., *Lactococcus garvieae* | 39/40(98%) | 9.00E-19 | 40 | BAK58604.1 |
| 46 | + | 35235 | 35831 | 198 | 22 | 5.5 | AGGAGctggtg**ATG** | Histidine phosphatase superfamily | H.p., *Lactococcus garvieae* | 196/198(99%) | 8.00E-146 | 198 | WP_019335713.1 |

^a^Orientation of the gene in the genome.

^b^MM, molecular mass.

^c^RBS, ribosomal binding site. The uppercase letter represent the hypothetical RBS sequences and in bold the starting codon, whereas the lowercase letter represent the nucleotides between the RBS sequence and the starting codon and also nucleotide after the RBS.

^d^ – indicates no significant matches.

^e^BLATp result corresponds to second best alignment.

^f^Total size of the aligned protein
